# Effects of resistance and functional-skills training on habitual activity and constipation among older adults living in long-term care facilities: a randomized controlled trial

**DOI:** 10.1186/1471-2318-6-9

**Published:** 2006-07-31

**Authors:** Marijke JM Chin A Paw, Mireille NM van Poppel, Willem van Mechelen

**Affiliations:** 1 Department of Public and Occupational Health, EMGO Institute VU University Medical Center Van der Boechorststraat 7 1081 BT Amsterdam, The Netherlands

## Abstract

**Background:**

Large-scale RCTs comparing different types of exercise training in institutionalised older people are scarce, especially regarding effects on habitual physical activity and constipation. This study investigated the effects of different training protocols on habitual physical activity and constipation of older adults living in long-term care facilities.

**Methods:**

A randomized controlled trial with 157 participants, aged 64 to 94 years, who were randomly assigned to 1) resistance training; 2) all-round functional-skills training; 3) both; or 4) an 'educational' control condition. Habitual physical activity was assessed with a physical activity questionnaire and accelerometers. Constipation was assessed by a questionnaire. Measurements were performed at baseline and after six months of training.

**Results:**

At baseline the median time spent sitting was 8.2 hr/d, the median time spent on activity of at least moderate intensity was 32 min/d. At baseline, about 22% of the subjects were diagnosed with constipation and 23% were taking laxatives. There were no between-group differences for changes in habitual physical activity or constipation over 6-months.

**Conclusion:**

Six months of moderate intensity exercise training neither enhances habitual physical activity nor affects complaints of constipation among older people living in long-term care facilities.

## Background

Physical activity is an important element of a healthy lifestyle. In general, the total amount of physical activity, as well as the diversity in types of activity, declines with age. In the Netherlands in 2000, 59% of people aged 65 and over were not engaged in sufficient physical activity to obtain health benefits, i.e. at least 30 minutes of moderate intensity activity on five days a week [1,2]. Older people living in long-term care facilities are the most inactive. Participation in exercise programs may enhance participation in habitual physical activity by improving the ability to better perform tasks of daily living and by improving enjoyment in physical activity. Additionally, increased social contacts may stimulate habitual physical activity. So far, few randomized controlled trials examined changes in habitual activity levels of older people [3-6].

Another possible benefit of exercise programs or enhancement in habitual physical activity may be a decline in complaints of constipation. Underlying mechanisms regarding the association between physical exercise and constipation are unclear but a favourable effect on colonic motility, decreased blood flow to the gut, biomechanical bouncing of the gut during running, compression of the colon by abdominal musculature, and increased fibre intake as a result of increased energy expenditure have all been reported [7]. Constipation is a common complaint in older people, and many people become gradually more constipated with age [8]. One of the contributing factors may be decreased mobility and physical inactivity. There is a paucity of controlled trials regarding the association between constipation and exercise in older people [6,9]. However, the Nurses' Health Study [10], which followed a cohort of 62,036 women, found that physical activity 2–6 times per week was associated with a 35 percent lower risk of constipation. The purpose of this study was, therefore, to evaluate the effects of three different group-based moderate-intensity exercise protocols on habitual physical activity and constipation among older people living in long-term care facilities.

## Methods

### Study design

We conducted a 6-months randomized controlled trial of three different moderate-intensity training protocols among older adults living in long-term care facilities, i.e. homes for the aged, with services ranging from independent living to skilled nursing. In this report we describe the effects on habitual physical activity and constipation, which were secondary outcome measures. Effects on the primary outcomes i.e. physical function and wellbeing are described elsewhere [11,12]. In each of the six homes, subjects were randomly assigned to one of the three exercise conditions or the control condition which was an 'educational' program. The random allocation sequence was generated by computer by two independent students, who also assigned participants to their group. Random assignment took place after completion of baseline measurements. The study protocol was approved by the VU University medical center ethics committee.

### Study population

Participants of the study were living in six residential and extended care facilities in the North-Western part of The Netherlands (i.e. West-Friesland). Informative meetings were organized in each home so that the design of the study and the interventions could be explained in detail. Information regarding the study was also available in brochures for the people to take home. At the end of the meeting, subjects received a form on which they could indicate whether they were interested in the study. Those interested in study participation were screened for eligibility based on the following inclusion criteria: 1) aged 65 or older; 2) living in a nursing home or residential care facility; 3) able to walk 6 m or more (with or without a walking aid); 4) able to comprehend the study procedures; 5) no medical contraindication for study participation; 6) no rapidly progressive or terminal illness; 7) and not moving away from the home within the 6-months intervention period (five and six were evaluated by their general practitioner). Informed consent was obtained from all subjects.

### Interventions

#### Resistance training

The resistance training program was performed twice a week during six months in groups of five to seven participants directed by a trained physical therapist and an assistant. In the first two weeks, participants were familiarized with the equipment and the technique of the exercises by exercising with minimal resistance. The following weeks, resistance increased until two sets of 8–12 repetitions were possible. Resistance was to be increased after the participant could complete two sets of 12 repetitions for two consecutive sessions. As a warm-up, each exercise was first performed 10–20 repetitions with minimal resistance. Progression was monitored with exercise logs filled out by the supervising physical therapist and assistant. The five exercises were leg press, lattisimus pulldown, biceps curl and triceps press on TechnoGym equipment, and heel raises with dumbbells (1 to 5 kg each), ankle and/or wrist weights (1 and 2 kg per pair). For the heel raises the number of repetitions were increased if the subjects could lift the maximum weight (2 × 5 kg dumbbells + 2 × 2 kg ankle weights). Sessions lasted 45–60 min and closed with stretching exercises. The program was designed to improve muscle strength of major muscle groups of both upper and lower body, important for functional performance on common daily activities, according to the American College of Sports Medicine recommendations for older adults [13].

#### Functional-skills training

The functional-skills training program was performed twice a week during six months in groups of 7–15 participants, directed by a trained physical therapist and an assistant. The first week was to familiarize participants with the technique of the exercises. All classes started with 5–10 min of warm-up activities: walking (whenever possible), exercise-to-music routines, becoming familiar with the equipment. This was followed by 30–35 min of skills training in game-like and cooperative activities, such as throwing and catching a ball while standing up and sitting down on a chair, musical chairs and team pursuit races. The cool-down period (5–10 min) consisted of stretching and relaxation activities (e.g. finger and wrist rolls, shoulder rolls, reaching, leg stretches). All exercises were adjusted to the individual mobility level. The intensity was gradually increased: the number of repetitions increased, exercises were performed more often standing up straight and the use of wrist and ankle weights (1 and 2 kg per pair) was encouraged. The program was designed to improve muscle strength, speed, endurance, coordination and flexibility thereby improving functional performance of common daily activities. An emphasis was placed on skills training, meaning that the specific activities required for independence in daily activities were practiced. The design and theoretical background of the functional-skills training program are described in detail elsewhere [14].

#### Combination

Subjects in the combination group performed once weekly the resistance training and once weekly the all-round functional-skills training protocol. All three exercise programs were directed by physical therapists who were already working in the homes and thus familiar with working with the study population. The physical therapists were trained by the principal investigator (MCAP). To encourage standardization, the training protocols were extensively described in a manual. The assistants were either volunteers or students.

#### Control program

The control program was designed to provide attention, social interaction and was meant to be a 'placebo' intervention. Participants were told that they were assigned to an 'educational' program (i.e. group discussions about topics of interest to older people such as history of the 20th century, music, relaxation etc.). Sessions were organized two days of the week during six months for 45–60 min in groups of 7–15 participants, supervised by a professional creative therapist.

### Measurements

Data were collected at baseline and after six months intervention by three trained research assistants who were blinded to group assignment, according to a standardized protocol.

### Physical activity

The level of physical activity was estimated by an accelerometer and the validated Lasa Physical Activity Questionnaire [15]. The questionnaire was administered in a personal interview at the subjects home. This questionnaire addresses the following activities: walking outdoors, bicycling, light household activities, heavy household activities, and a maximum of two sports activities. Respondents were asked how often and for how long in the previous two weeks they had engaged in each activity. From the questionnaire data, two measures of total physical activity were calculated. First, activity in min/d was calculated by multiplying the frequency and duration of each separate activity in the previous two weeks and dividing the multiplied score by 14. A total activity score was calculated by summing all separate activity scores. The total time spent on moderate-intensity physical activity was calculated by subtracting the time spent on light household activity from the total activity time.

The MTI model 7164 accelerometer was worn on a belt around the hip during three consecutive days. The accelerometer measures acceleration and deceleration in a vertical plane over a user-specified interval (epoch). The accelerometer was initialized as described by the manufacturer and the 60-s epoch was used. The subjects were carefully instructed on how to wear the accelerometers for the entire awake time of the day, except during water activities. From the accelerometer data three measures of physical activity were calculated: the average time spent sitting per day using an arbitrary cut off (< 100 counts/min); the average total counts per day over the registered days; and the average number of counts per minute (total counts divided by the registration time).

### Bowel movements

Subjects were asked about a number of defecation problems and frequency of bowel movements. They were also asked about the use of laxatives (including synthetic laxatives, bulk forming organic laxatives and enemas). For the diagnosis of constipation, a subject must have had two or more complaints, but not taking laxatives. Complaints were defined as: straining at least 25% of the times, lumpy or hard stools at least 25% of the times, a feeling of incomplete evacuation at least 25% of the times, and fewer than three bowel movements in a week. This definition of constipation is an adaptation of that described by Schaefer & Cheskin [8] who used fewer than two bowel movements per week as a criterion.

### Statistical analysis

Data were analyzed using SPSS for Windows (release 7.5.2) and MlwiN (1998, version 1.02.0002). Descriptive data are reported for variables of interest (percentage, mean, SD). Analysis of variance and ϖ^2^-tests were used to compare groups at baseline. To evaluate the effects of the interventions multilevel analysis was used. Using this technique, regression coefficients can be adjusted for the clustering of observations within one home that leads to dependency of observations of different subjects within one home. In the multi-level analysis two levels were defined: 1) patient and 2) home. A linear model was used to study the effect of all three interventions on the continuous outcome values. The parameters of interest are the regression coefficients (beta) indicating the effect of the intervention of interest, compared to the control group. In the 'crude' model the outcome value at six months was adjusted only for the value at baseline. In the secondary analyses, adjustments were made for gender, age and class attendance. Regression coefficients and 95% confidence intervals for the basic and the adjusted model are reported.

The trial was designed to randomize 60 subjects to each intervention group, taking into account a dropout percentage of 25% with an alpha of 0.05 and a power of 0.80. Primary analyses were conducted by intention-to-treat with participants analyzed according to the initial randomized assignment.

## Results

In total, 224 subjects were enrolled in the study and randomly assigned to the four intervention groups. Mean age of these subjects was 81.7, ranging from 64 to 94 yr. For 157 subjects (70%) complete data were available from the physical activity questionnaire. Mainly due to technical problems, complete accelerometer data were available for 118 subjects (53%). General characteristics of this group are shown in Table 1. Their mean age was 81 (± 5.6) yr and the majority lived in a residential care facility. Dropout of participants was not significantly different among the four groups (resistance training 30%, functional-skills training 27%, combined training 21% and control group 39%). The study participants who dropped out were slightly older (83 versus 81 yr), and were more often male (20 versus 15%) and living in nursing homes (23 versus 14%).

**Table 1:** General characteristics of elderly living in long-term care facilities (N = 157)

	**Resistance training (n = 40)**	**Functional training (n = 41)**	**Combined training (n = 45)**	**Control (n = 31)**
Age, mean ± SD, yr	81.0 (± 5.8)	82.1 (± 4.9)	80.9 (± 6.3)	81.3 (± 4.4)
Sex, No. (%) male/female	11(27)/29(73)	8(20)/33(80)	7(16)/38(84)	5(16)/26(84)
marital status, No. (%)				
- *Married*	8 (20)	7 (17)	10 (22)	11(36)
- *Widowed*	28 (70)	30 (73)	32 (71)	19 (61)
- *never married*	1 (3)	2 (5)	3 (7)	1 (3)
- *divorced*	3 (8)	2 (5)	-	-
type of residence, No. (%)				
- *nursing home*	6 (15)	5 (12)	7 (16)	2 (7)
- *residential care*	34 (85)	36 (88)	38 (84)	29 (93)

Median attendance to the resistance training was 76%, to the functional-skills training 70% and to the combined training 73%. Attendance to the control program was significantly lower (67%). No participant withdrew for adverse effects, but eight participants discontinued the intervention because they found the exercise program too intensive.

### Physical activity

Physical activity characteristics at baseline and after six months are presented in Table 2. At baseline, the median time spent on physical activity was almost 105 min/d, and the median time spent sitting 8.2 h/d. The median time spent on activity of at least moderate intensity was 32 min/d. About half (47%) of the study sample spent less than 30 min/d (the recommended amount of physical activity in the Netherlands) on moderate intensity activities. Mean accelerometer counts per day were 103,647 and the number of counts per minute 133. There were no differences between groups at baseline, except that the resistance training group spent less time on total physical activity. These values did not change significantly after six months intervention.

**Table 2:** Baseline physical activity and 6-month change (median [25th and 75th percentile] or mean ± sd) of elderly living in long-term care facilities (N = 157)

	**Resistance training**		**Functional training**		**Combined training**		**Control**	
	(n = 40)		(n = 41)		(n = 45)		(n = 31)	
	baseline	6 m change	baseline	6 m change	baseline	6 m change	baseline	6 m change
	
total physical activity (min/d)	85 [47;120]*	12 [-20;39]	118 [71;178]	-2 [-43;36]	111 [64;167]	1 [-31;22]	128 [69;159]	-10 [-39;30]
total moderate activity (min/d)	21 [6.41]	-6 [-22;6]	41 [18;59]	-3 [-22;6]	43 [9;52]	-4 [-17;3]	32 [11;81]	1 [-17;15]
Time spent sitting (min/d)	508 [204;568]	-3 [-17;41]	484 [277;579]	-8 [-58;29]	510 [305;569]	8 [-56;64]	466 [214;532]	0 [-31;19]
total counts per day	87778 ± 37427	7397 ± 25631	102137 ± 45412	-455 ± 44344	119460 ± 66204	-528 ± 49601	105610 ± 48437	2808 ± 40467
counts per minute	113 ± 45	7 ± 29	133 ± 54	-0.1 ± 49	150 ± 77	1 ± 66	136 ± 58	3 ± 40

* p ≤ 0.05, for between group difference at baseline

**Figure 1:**
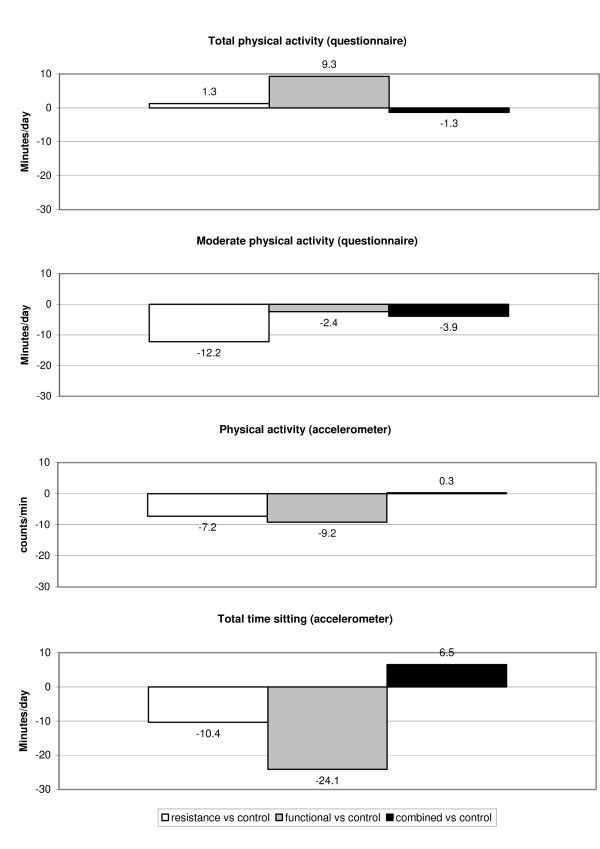
Mean difference in 6-month change in habitual physical activity between exercise groups and the control group.

The differences in the 6 months change between the three exercise groups compared to the control group are presented in Table 3 and Figure 1. After adjusting for score at baseline, age, sex and class attendance no significant differences between the exercise groups and controls were found, except for a decline in activity of at least moderate intensity in the resistance training group compared to the controls (adjusted difference: -12.2 min, 95% CI:-23.8;-0.7). Both the resistance training and the combined training group spent less time sitting compared to the control group after 6 months intervention. However, this difference was not statistically significant.

**Table 3:** Results of Multilevel analyses regarding the effect of three different training protocols on habitual activity of elderly living in long-term care facilities (N = 157)

	**Resistance training versus control **difference [95% CI]	**Functional training versus control **difference [95% CI]	**Combined training versus control **difference [95% CI]
total physical activity (min/d)			
adjusted for score at baseline	3.9 [-21.9;29.7]	9.6 [-15.4;34.6]	2.8 [-21.7;27.3]
adjusted for score at baseline, age, sex and compliance	1.3 [-24.2;26.7]	9.3 [-15.4;33.9]	-1.3 [-25.9;23.3]
total moderate activity (min/d)			
adjusted for score at baseline	-11.5 [-23.0;0.04]	-2.4 [-13.6;8.9]	-3.18 [-14.2;7.9]
adjusted for score at baseline, age, sex and compliance	-12.2 [-23.8;-0.7]*	-2.4 [-13.7;8.9]	-3.9 [-15.2;7.4]
averaged time spent sitting (min/d)			
adjusted for score at baseline	-6.3 [-50.2;37.7]	-19.1 [-62.7;24.6]	4.4 [-39.6;48.3]
adjusted for score at baseline, age, sex and compliance	-10.4 [-54.0;33.2]	-24.1 [-67.3;19.1]	6.5 [-37.3;50.3]
total counts per day			
adjusted for score at baseline	-2471 [-22106;17164]	-5068 [-24413;14277]	3466 [-16146;23078]
adjusted for score at baseline, age, sex and compliance	-5939 [-25531;13653]	-8891 [-28105;10323]	202 [-19496;19900]
counts per minute			
adjusted for score at baseline	-3.4 [-28.3;21.5]	-5.0 [-29.6;19.5]	4.1 [-20.7;29.0]
adjusted for score at baseline, age, sex and compliance	-7.2 [-32.2;17.9]	-9.2 [-33.8;15.3]	0.3 [-24.9;25.4]

* p ≤ 0.05, for exercise versus control group

### Bowel movements

For 172 subjects (77%) complete data were available from the questionnaire on bowel movements. No differences in the number of subjects with constipation or taking laxatives were observed between groups at baseline. About 22% of the subjects were diagnosed with constipation and 23% were taking laxatives at baseline. No statistically significant differences between men and women and type of residence were present at baseline. Neither of the exercise programs had an effect on the percentage of subjects with constipation or taking laxatives.

## Discussion

This study demonstrates that twice-weekly moderate-intensity exercise programs affects neither habitual physical activity nor constipation in habitants of long-term care facilities. We hypothesized that participation in exercise programs may improve habitual physical activity by improving the ability to perform tasks of daily living, enjoyment in physical activity, and increased social contacts.

One of the reasons for the lack of effect on habitual physical activity may be that subjects were insufficiently compliant to improve their ability to perform tasks of daily living. Twice weekly functional-skills training, or a combination of resistance and functional-skills training did improve several fitness and performance measures, while exercising once a week appeared to be insufficient [11]. Even in a more compliant subgroup who attended at least 75% of all exercise classes no enhancement of habitual physical activity was noted [11]. Looking at the resistance exercise logs all supervising physical therapists frequently modified the resistance training protocol in case subjects could not perform the 8–12 repetitions without complaints. Both our participants as well as the supervising physical therapists were reluctant to increase the resistance. Therefore, the increase in resistance often was at a slower pace than according to the protocol. Adherence to the functional-skills training protocol is more difficult to assess. Since twice weekly functional-skills training alone or in combination with resistance training did show functional improvement, functional-skills training seems more feasible in this type of population. On the other hand, the majority of subjects did enjoy participation in the exercise programs and wanted to continue exercising after the 6-month trial.

Our findings are contradictory with the findings of Hamdorf et al. [5] and Fiatarone et al. [4]. Hamdorf et al[5] found that habitual activity patterns (according to the Maximum Current Activity 'still doing' and Normative Impairment Index) had increased among 80 healthy women aged 60 to 70 after a 26 wk progressive walking program. Fiatarone et al. [4] reported that overall level of physical activity (assessed by accelerometers worn around both ankles) increased among 45 frail nursing home residents after 10 wk progressive resistance exercise training. However, the level of physical activity in the preceding studies was assessed differently and less elaborately. Possibly for a frail population another type of exercise intervention may be more successful in increasing physical activity. In the study of Simmons and Schnelle [6] research staff provided exercise and toileting assistance every two hours, four times per day, five days a week for 32 weeks, and this did result in overall physical activity increases but not in improved bowel movement. One should bear in mind that habitual physical activity is difficult to assess and there is no perfect method. We used a physical activity questionnaire and an accelerometer to assess habitual physical activity subjectively and objectively. Results from both methods suggest that subjects had not increased their level of physical activity.

According our expectations the study population was extremely inactive. The physical activity questionnaire was also used in the Longitudinal Aging Study Amsterdam (LASA). Compared to participants of LASA (n = 2,109), who were younger (69.3 yr) and independently living, our population was less physically active (2 h/d vs 3 h/d) [16]. The amount of physical activity according to the accelerometer was much smaller compared to healthy adults (133 counts/min versus 402 counts/min) [17]. Also, about half of the study population were not sufficiently active according to the Dutch recommendation regarding the minimum physical activity time required to enhance health. This finding was based on subjective questionnaire data and comparable to prevalence data in the general adult population [2]. It suggests that subjects in our study tended to overestimate or over-report their amount of physical activity. To calculate how much time subjects spent in different intensities of activity from the objective accelerometer data, cut-points are needed. At present, there exist different cut-points in the literature, but these are mainly derived from laboratory studies in healthy adults. For this reason we decided to calculate from the accelerometer data only the total counts over the registered days, and the number of counts per minute, and not the total time spent on moderate intensity physical activity. The time spent sitting was calculated using an arbitrary cut-off point of 100 counts/min.

The percentage of subjects with constipation in our study (22% according to our definition) was low for this age group. Earlier studies reported a prevalence of constipation as high as 26% among men and 34% among women over 65 yr [8]. The use of laxatives was remarkably low also (23%). An earlier study reported that about 56% of nursing home residents used laxatives for bowel regulation in the Netherlands [2,6,18]. A possible explanation may be that the questions we used to assess constipation were not valid. The low prevalence of constipation in our sample may explain the lack of effect on complaints of constipation or the use of laxatives. Our finding agrees with those of Meshkinpour et al. [9] who studied the effects of regular exercise in the management of constipation among patients with chronic idiopathic constipation in the age range of 31 to 65 yr. Simmons and Schnelle [6] found that daily exercise in addition to a scheduled-toileting intervention were not sufficient to improve bowel movement frequency. Constipation is a multi faceted problem that is thought to be related to physical activity, food and fluid intake and medications. In older people physical activity may help in improving constipation but probably as part of a broad intervention that affects all of these risk factors.

A strength of this study is that instead of an efficacy study, we meant to perform an effectiveness study evaluating the relative effects of different exercise protocols on habitual physical activity and complaints of constipation function of long-term care residents under more 'real life' circumstances. Exclusion criteria for study participation were kept to a minimum and the exercise programs were supervised by physical therapists who already worked in the long-term care facilities. We realize that supervision of the programs by research staff might have improved standardisation and adherence to the exercise protocols. However, supervision by facility staff as in our study resembles more the real-life situation.

A limitation of our study is that because of the nature of the trial (i.e. an effectiveness trial rather than an efficacy trial), there was no close control or exact measurement of the exercise intensity by the researchers. Other limitations are the fact that complaints of constipation were assessed by a short questionnaire and that no information was available on possible changes in food- and water intake and use of medication. More research is needed on implementation of exercise programs in home-based settings and their effectiveness on habitual physical activity and constipation. Practical systems for monitoring exercise intensity in care settings are needed. According to Glasziou et al. [19] monitoring should aim to establish the response to the exercise intervention, detect the need to adjust the intervention and detect adverse effects. Since constipation is a multi-faceted problem, future research on this common complaint should include not only physical activity, but also food and fluid intake and medication use.

## Conclusion

Six months of moderate intensity exercise training neither enhances habitual physical activity nor affects complaints of constipation among older people living in long-term care facilities.

## Competing interests

The author(s) declare that they have no competing interests.

## Authors' contributions

MCAP conceived of the study, participated in its design and coordination, performed the statistical analysis and drafted the manuscript. MvP participated in the coordination of the study and the statistical analysis. WvM conceived of the study and participated in the design of the study. All authors read and approved the final manuscript.

**Table 4:** The number of subjects (%) with constipation or taking laxatives among the four research groups at baseline and six months follow-up (N = 172).

	**Strength training ** (n = 41)		**Functional training ** (n = 48)		**Combined training ** (n = 48)		**Control ** (n = 35)	
	baseline	6 mo	baseline	6 mo	baseline	6 mo	baseline	6 mo
	
No. (%) of subjects with constipation*	9/41 (22)	9/41 (22)	9/48 (19)	11/48 (23)	12/48 (25)	14/48 (29)	8/35 (23)	3/35 (9)
No (%) of subjects using laxatives								
- daily	4/41 (10)	5/41 (12)	8/48 (17)	5/48 (10)	8/48 (17)	9/48 (19)	9/34 (26)	5/35 (14)
- 2–3 d/wk	2/41 (5)	1/41 (2)	1/48 (2)	0/48 (0)	1/48 (2)	0/48 (0)	1/34 (3)	0/35 (0)
- weekly	1/41 (2)	2/41 (5)	0/48 (0)	2/48 (4)	2/48 (4)	1/48 (2)	0/34 (0)	1/35 (3)
- never	34/41 (83)	33/41 (80)	39/48 (81)	41/48 (85)	37/48 (77)	38/48 (79)	24/34 (71)	29/35 (83)

* Subjects were considered to be constipated if they were not taking laxatives and had two or more of the following complaints: straining, lumpy or hard stools, feeling of incomplete evacuation, fewer than three bowel movements in a week.

## Pre-publication history

The pre-publication history for this paper can be accessed here:


